# The effect of compressive trabecular bone-cephalocervical implant relationship on stability in intertrochanteric femoral fractures: a clinical review and biomechanical research

**DOI:** 10.3389/fbioe.2025.1628529

**Published:** 2025-09-01

**Authors:** Lincong Fei, Jinhui Liu, Liang Zhao, Can Mou, Wei Fang, Junwu Ye

**Affiliations:** ^1^ Department of Orthopaedics I, Traditional Chinese Medicine Hospitalof Meishan, Meishan, China; ^2^ Department of Orthopedics, The Affiliated Hospital of Southwest Medical University, Luzhou, China; ^3^ Sichuan Provincial Laboratory of Orthopaedic Engineering, Luzhou, China

**Keywords:** intertrochanteric femoral fractures, compressive trabecular bone-cephalocervical implant relationship, stability, clinical review, biomechanical research

## Abstract

**Introduction:**

Compressive trabecular bone plays a vital role in load transmission within the proximal femur, and regional variations in cancellous bone distribution have been shown to affect implant stability. However, the biomechanical influence of the spatial relationship between compressive trabecular bone and cephalocervical implants on postoperative fracture stability in intertrochanteric femoral fractures remains unclear.

**Methods:**

In this study, we conducted a retrospective analysis of 64 patients treated with proximal femoral nail antirotation (PFNA) Lever arm parameters reflecting the spatial relationship between compressive trabecular bone and the cephalocervical implant were measured on initial postoperative anteroposterior and lateral radiographs, while the zonal classification of the implant relative to the trabecular architecture was assessed to evaluate its impact on early femoral head varus and helical blade displacement. Additionally, seven finite element models with different implant positions were established to investigate the biomechanical mechanisms underlying stability.

**Results:**

The results indicated that, a larger trabecular bone-implant lever arm and lower bone mineral density (BMD) independently increased the risks of femoral head varus (*p* < 0.01) and blade displacement (*p* < 0.01). Positioning the implant within Zone C of the trabecular architecture was associated with reduced incidences of femoral head varus and implant displacement (*p* < 0.05). Biomechanical analysis further demonstrated that placing the implant in Zone C with minimized lever arm resulted in the smallest femoral head varus, blade displacement, and the least apparent stress concentration at the implant tip within cancellous bone.

**Discussion:**

These findings suggest that intraoperative placement of cephalocervical implants should aim to reduce the trabecular bone-implant lever arm and prioritize positioning within Zone C of the trabecular architecture to enhance early stability. However, further validation through comprehensive finite element analyses and biomechanical experiments is required.

## 1 Introduction

The overall incidence of hip fractures in individuals over 60 years reaches 2.36% (Ren et al., 2019), with a persistently increasing trend. It is estimated that the number of hip fractures in Asia will rise to 2.28 times the 2018 level by 2050 (Cheung et al., 2018). Intertrochanteric fractures account for approximately 40% of hip fractures (Adeyemi and Delhougne, 2019), and early surgical fixation remains the primary treatment (Fischer et al., 2021). However, implant cutout continues to be a critical factor compromising clinical outcomes (Wu, LF et al., 2024). Most cutouts occur in the cephalocervical fragment (Garabano, G et al., 2024), typically stabilized by helical blades or lag screws—collectively termed “cephalocervical implants” in this study. Implant positioning significantly influences fixation stability. Current consensus (Haidukewych, 2010) suggests optimal placement when the tip-apex distance (TAD) is <25 mm, with the implant tip located in the central or central-inferior quadrant of the femoral head. Paradoxically, inferior implant positioning may reduce cutout rates despite increasing TAD (De Bruijn et al., 2012), and discrepancies in TAD reliability due to variations in femoral head size (Subasi et al., 2022) have sparked ongoing debates regarding ideal implant placement.

Numerous evaluation systems exist for assessing cephalocervical implant positioning. Early methods include the Cleveland femoral head dividing system (Cleveland et al., 1959), Parker ratio (Parker, 1992), TAD (Baumgaertner et al., 1995), and calcar-referenced TAD (CalTAD) (Kuzyk et al., 2012). Recent innovations encompass the axis-blade angle (ABA) (Mao et al., 2019), tip-neck distance ratio (TNDR) (Çepni et al., 2022), and standardized TAD (STAD) (Yang Y. F. et al., 2023). While these methods derive from biomechanical studies and clinical observations, the biomechanical significance of the proximal femoral trabecular architecture—particularly compressive trabeculae—in guiding implant positioning remains unexplored.

Local bone quality modulates stress distribution at the bone-implant interface, potentially altering stress concentration patterns and load transfer mechanisms that govern immediate postoperative stability (Zhang et al., 2023). The proximal femur features distinct rabecular arrangements, where compressive trabeculae predominantly mediate mechanical load transmission. Theoretically, variations in implant-trabecular spatial relationships could differentially influence biomechanical behavior, yet no published studies have systematically investigated how compressive trabecular-implant interactions affect fixation stability.

Building upon these theoretical and clinical foundations, we hypothesize that spatial relationships between compressive trabeculae and cephalocervical implants critically modify postoperative biomechanical environments in intertrochanteric fracture fixation. To test this hypothesis, we conducted a clinical retrospective study and developed seven finite element models with varying trabecular-implant spatial configurations. Through these investigations, we demonstrate the biomechanical rationale for optimizing implant positioning relative to compressive trabecular architecture, providing new insights into cephalocervical implant placement strategies to enhance fixation stability and clinical outcomes.

## 2 Materials and methods

### 2.1 Clinical review

#### 2.1.1 Study subjects and inclusion/exclusion criteria

**FIGURE 1:**
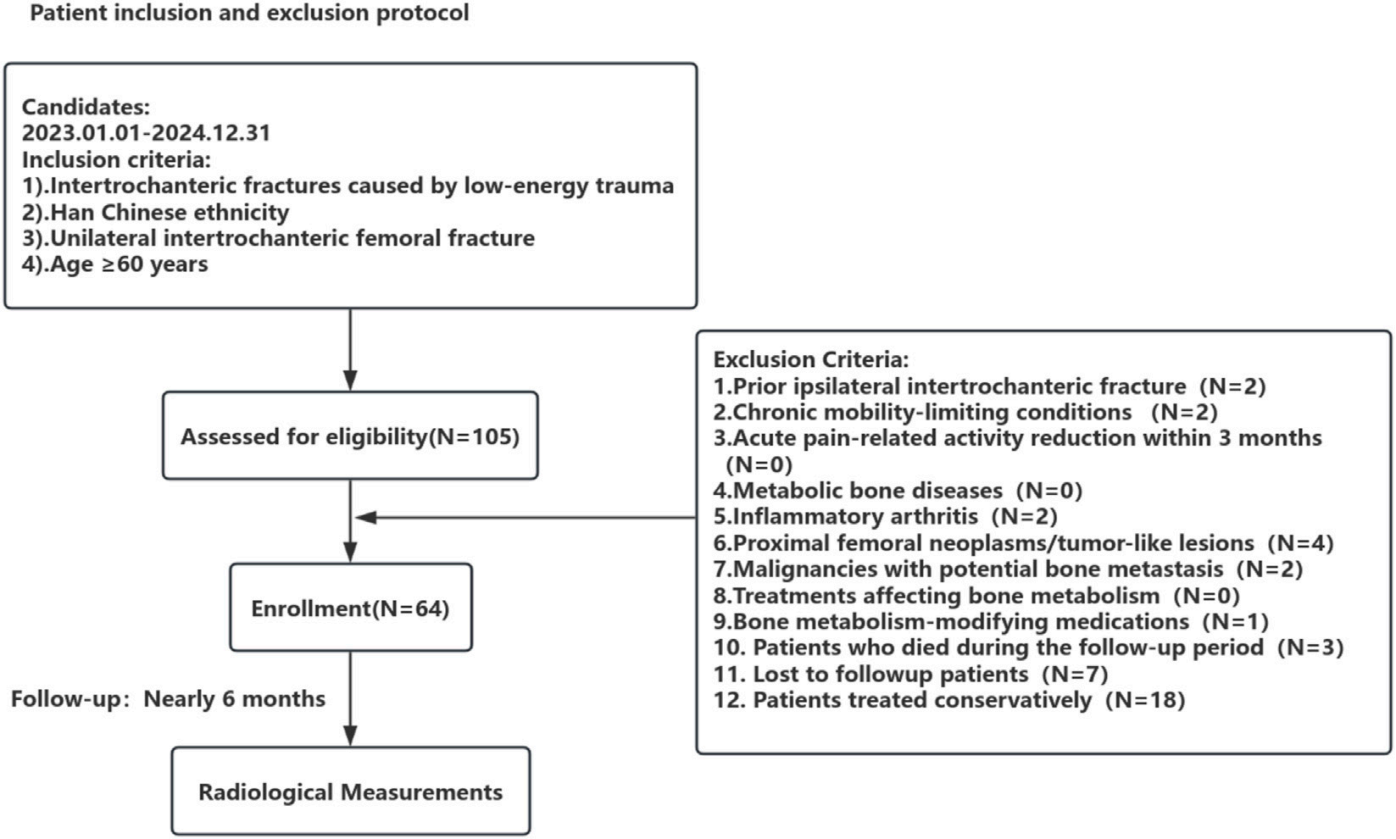
Patient inclusion and exclusion protocol.

This study received ethical approval from Southwest Medical University (Approval No. 20240522-001). We retrospectively enrolled elderly patients with intertrochanteric femoral fractures treated by closed/limited open reduction and internal fixation using proximal femoral nail antirotation (PFNA) at our institution between January 2023 and December 2024 (Figure 1).

Inclusion Criteria: 1. Intertrochanteric fractures caused by low-energy trauma; 2. Han Chinese ethnicity; 3. Unilateral intertrochanteric femoral fracture; 4. Age ≥60 years.

Exclusion Criteria: 1. Prior ipsilateral intertrochanteric fracture; 2. Chronic mobility-limiting conditions (e.g., paralysis, malunited lower extremity fractures, hip dysplasia, avascular necrosis of the femoral head); 3. Acute pain-related activity reduction within 3 months (e.g., acute pancreatitis, vertebral fractures); 4. Metabolic bone diseases (excluding senile or postmenopausal osteoporosis); 5. Inflammatory arthritis (e.g., rheumatoid arthritis); 6. Proximal femoral neoplasms/tumor-like lesions (e.g., bone metastases, chondrosarcoma, bone islands); 7. Malignancies with potential bone metastasis; 8. Treatments affecting bone metabolism; 9. Bone metabolism-modifying medications (e.g., glucocorticoids); 10. Patients who died during the follow-up period; 11. Lost to followup patients; 12. Patients treated conservatively.

In summary, only low-energy unilateral intertrochanteric fracture patients of Han Chinese ethnicity were included. Individuals with preexisting mobility limitations, recent activity reduction, or conditions potentially affecting bone integrity were excluded. The final cohort comprised 64 patients (24 males, 40 females) with a mean age of 75.06 ± 9.57 years.

#### 2.1.2 Radiological measurements

**FIGURE 2:**
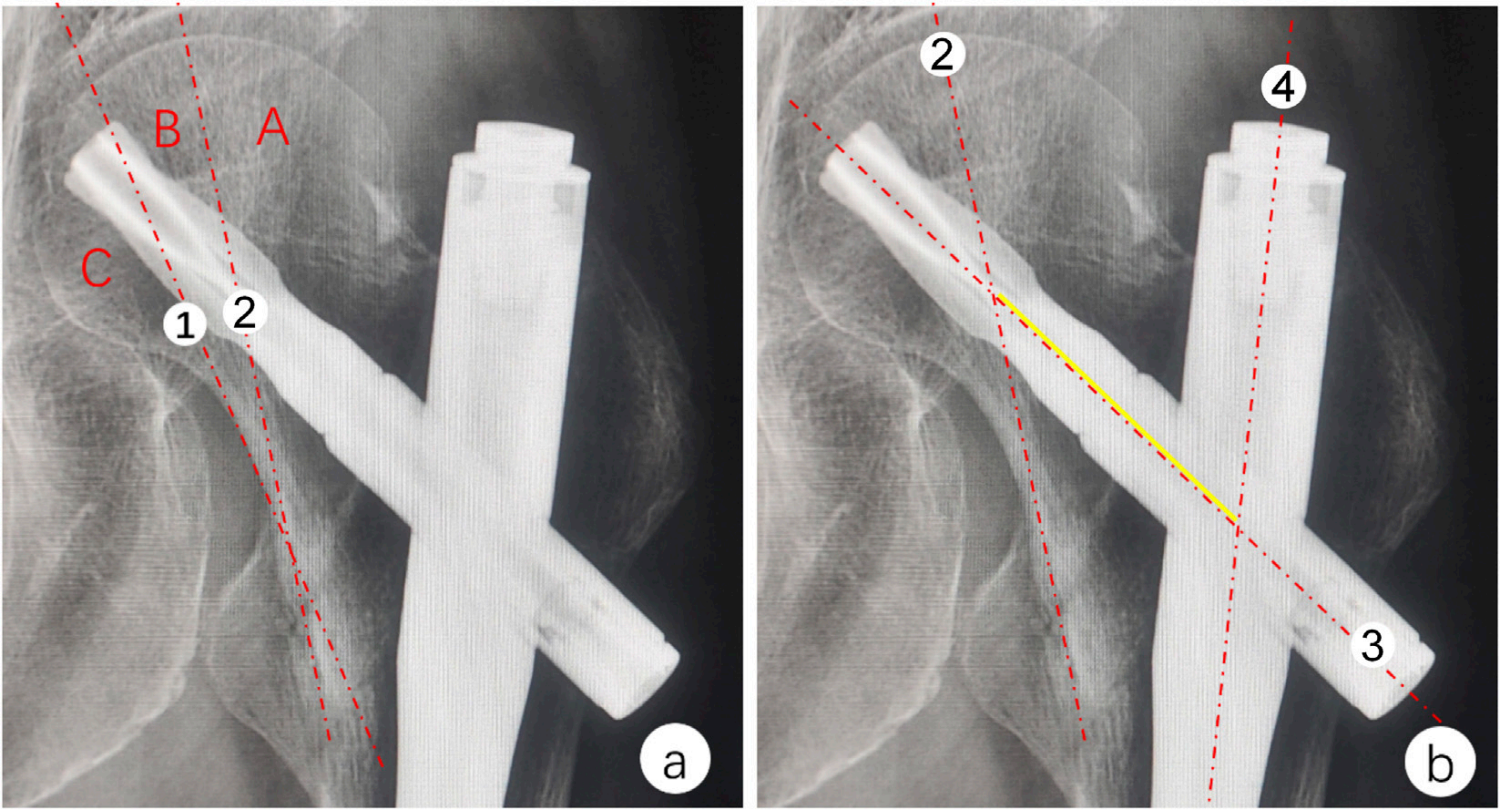
**(a)** Zoning diagram: Line 1: Inferior boundary of compressive trabecular bone; Line 2: Midline of compressive trabecular bone; Labeled zones: A: Superior to trabecular midline; B: Inferior to trabecular midline within trabecular region; C: Sub-trabecular region. **(b)** Schematic of trabecular-implant lever arm measurement:Line 3: Central axis of cephalocervical implant; Line 4: Central axis of main nail; Yellow arrow: Lever arm measurement between intersection points.

All radiological measurements were independently conducted by an orthopedic physician with extensive experience in orthopedic imaging interpretation. On immediate postoperative X-ray films, currently used evaluation parameters for cephalocervical implant positioning were measured, including tip-apex distance (TAD), calcar-referenced TAD (CalTAD), Parker ratio, axis-blade angle (ABA), and standardized tip-apex distance (STAD). On anteroposterior X-ray films taken immediately after surgery and at the 6-month follow-up, the neck-shaft angle of the affected limb and the relative displacement of the cephalocervical implant tip were measured. The difference in neck-shaft angle was calculated as the degree of femoral head varus (Nie et al., 2022). On immediate postoperative X-ray films, the spatial relationship between compressive trabecular bone and the cephalocervical implant proposed in this study was measured. Our research employed the following two parameters to evaluate this relationship (Figure 2):

Compressive Trabecular Bone Zoning: Based on prior literature (Liang et al., 2018), the femoral head region was divided into three zones using the inferior boundary of the compressive trabecular bone and the midline: midline superior zone (Zone A), midline inferior zone within the trabecular region (Zone B), and subtrabecular zone (Zone C).

Compressive Trabecular Bone-Implant Lever Arm: Defined as the distance from the intersection between the central axis of the cephalocervical implant and the central axis of the main nail to the intersection between the central axis of the cephalocervical implant and the midline of the compressive trabecular bone.

#### 2.1.3 Statistical analysis

Data were analyzed using SPSS 27.0 software. Normally distributed data are expressed as mean ± standard deviation, while non-normally distributed data are presented as median (P25, P75). Pearson correlation analysis (correlation coefficient denoted as *r*) was applied for normally distributed variables, with P < 0.05 indicating statistical significance. Spearman correlation analysis was used for non-normally distributed variables, calculating the regression coefficient *b* and corresponding P-value (P < 0.05 considered causally significant). Homogeneity of variance was assessed for compressive trabecular zoning groups using Levene’s test.

### 2.2 Biomechanical analysis

#### 2.2.1 Reconstruction of intertrochanteric fracture model

**FIGURE 3:**
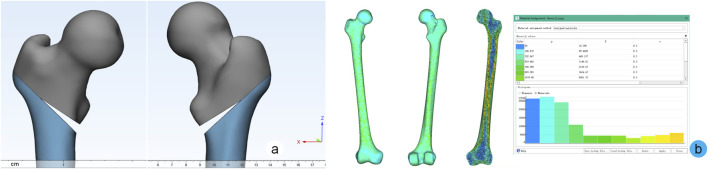
Establishment of fracture model and grayscale interval assignment process. **(a)** Anterior-posterior views of the AO/OTA 31-A2.1 type fracture model; **(b)** Simulation of heterogeneous bone density distribution in the femur through grayscale-based interval partitioning.

A right femoral full-length CT dataset (slice thickness 1.0 mm, axial continuous scanning) from a 20-year-old male in the hospital imaging database was selected. Model reconstruction was performed using Mimics 21.0 and Geomagic 2013. For material property assignment, we employed a validated grayscale-based interval partitioning method (Zhang et al., 2013), which outperforms traditional cortical/cancellous bone separation methods, to accurately simulate the heterogeneous distribution of proximal femoral cancellous bone. To construct the intertrochanteric fracture model, we referenced established methodologies (Goffin et al., 2013). In Geomagic 2013, partial intertrochanteric elements were removed to create an AO/OTA 31-A2.1 type fracture. The fracture line was oriented at 43° relative to the femoral shaft axis, positioned inferior to the lesser trochanter. A wedge-shaped fragment was generated by rotating the fracture line 10° clockwise, with the fragment depth occupying 60% of the fracture line length (Figure 3).

#### 2.2.2 Construction of PFNA fixation model

**FIGURE 4:**
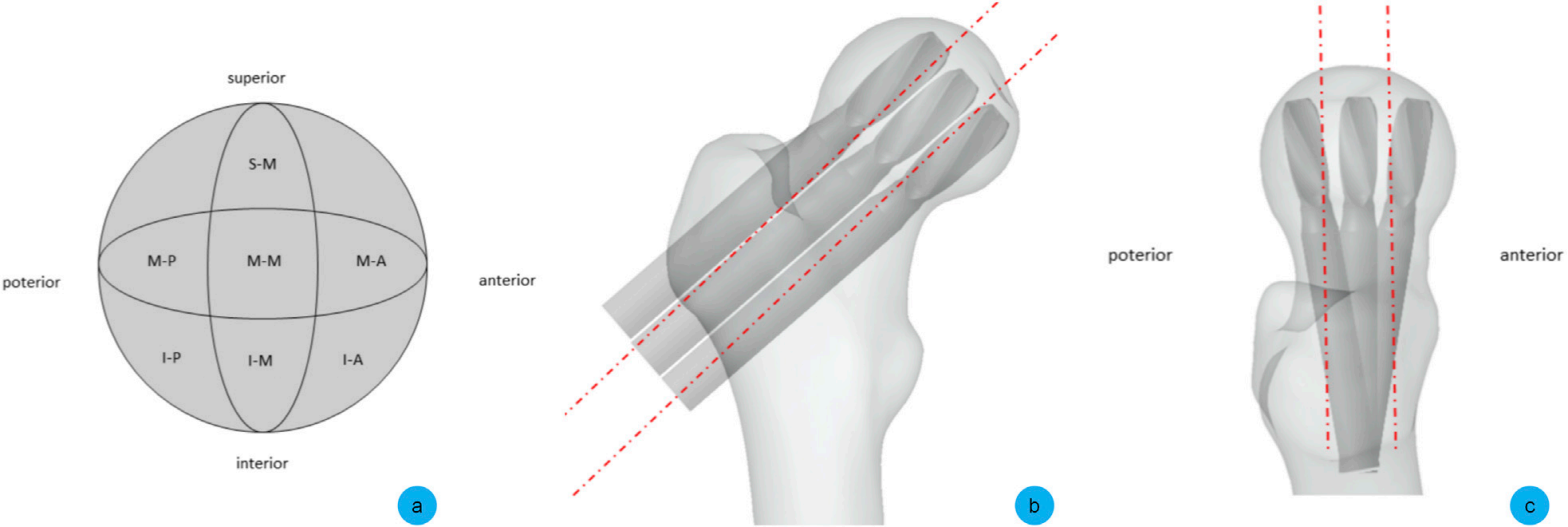
Schematic diagram of internal fixation assembly for intertrochanteric fracture. **(a)** Illustrations of experimental groups annotated on the femoral head zoning system; **(b)** Coronal view demonstrating bone-implant positioning in superior, middle, and inferior configurations; **(c)** Transverse view showing bone-implant positioning in anterior, middle, and posterior configurations.

Following surgical protocols and AO fracture fixation principles, the constructed AO/OTA 31-A2.1 intertrochanteric fracture model was assembled with a proximal femoral nail antirotation (PFNA) system in varying positions using assembly modules. Helical blades of identical insertion depth were implanted in seven distinct configurations: Superior-Medial (S-M); Mid-Medial (M-M); Mid-Anterior (M-A); Mid-Posterior (M-P); Inferior-Medial (I-M); Inferior-Anterior (I-A); Inferior-Posterior (I-P). The S-M, M-M, and I-M configurations specifically simulated positioning within Zone A, B, and C of the compressive trabecular architecture, respectively, while progressively reducing the trabecular-implant lever arm (Figure 4).

#### 2.2.3 Boundary and loading conditions

Numerical simulations for this study were carried out using “Ansys19.0”. The distal femur was fully constrained, neglecting the additional muscular forces present under physiological conditions. The coefficient of friction was set as follows: 0.46 for bone-bone interfaces, 0.3 for the bone-proximal femoral nail antirotation (PFNA) interface, and 0.23 for inter-component interfaces of the PFNA system (Eberle et al., 2010). A 20 mm^2^ loading surface was defined superior to the femoral head, applying a 670 N load (equivalent to 100% body weight) to simulate proximal femoral loading during standing. The load vector was oriented at 13° relative to the femoral mechanical axis in the anteroposterior direction and 8° posteriorly in the lateral plane, replicating physiological load transfer patterns. Peak femoral head displacement magnitude was recorded throughout the analysis to evaluate PFNA fixation stability and predict potential failure risks (Li, 2016).

## 3 Results

### 3.1 Correlation between various cephalocervical implant positioning evaluation methods and fixation stability

Correlation analysis revealed statistically significant associations between BMD, CalTAD, TAD, Parker ratio (AP), ABA, trabecular-implant lever arm, and neck-shaft angle changes, while vertical displacement correlated only with BMD, the trabecular-implant lever arm and ABA. Significant intergroup differences in vertical displacement and neck-shaft angle changes (0.7°–12.8°) were observed across compressive trabecular zones (*p* < 0.05), with Zone C demonstrating the smallest values (Table 1).

**TABLE 1:** Correlation coefficients between femoral head varus and variates.

Femoral head varus	Neck-shaft angle change		Vertical displacement	
	Correlation coefficients	*p*-value	Correlation coefficients	*p*-value
Sex (Male: 1, Female: 2)	0.049	0.747	0.141	0.355
Age	-0.125	0.414	-0.014	0.928
Side	-0.059	0.698	-0.256	0.089
BMD	0.423	0.004**	0.396	0.007**
TAD	-0.333	0.025*	-0.229	0.130
CalTAD	-0.339	0.023*	-0.215	0.157
Parker ratio (AP)	-0.306	0.041*	-0.226	0.135
Parker ratio (Lat)	-0.096	0.528	0.122	0.426
ABA	0.361	0.015*	0.391	0.008**
trabecular-implant lever arm	-0.497	<0.001**	-0.426	0.004**

*Statistical significance (p < 0.05).

**Statistical significance (p < 0.01).

Univariate regression analysis further identified BMD, ABA and trabecular-implant lever arm as independent risk factors for increased vertical displacement, whereas BMD and the trabecular-implant lever arm independently predicted reduced neck-shaft angle. No other parameters exhibited significant correlations with femoral head varus collapse and were not established as independent risk factors (Table 2).

**TABLE 2:** Linear regression analysis of femoral head varus.

Femoral head varus	Neck-shaft angle change		Vertical displacement	
	t	*p*-value	t	*p*-value
BMD	3.063	0.004**	2.832	0.007**
TAD	-0.196	0.846	-0.413	0.682
CalTAD	-0.538	0.594	-0.225	0.823
Parker ratio (AP)	0.327	0.745	1.281	0.208
Parker ratio (Lat)	-0.201	0.842	1.819	0.077
ABA	0.016	0.988	2.122	0.040*
trabecular-implant lever arm	-2.205	0.034*	-1.030	0.310

*Statistical significance (p < 0.05).

**Statistical significance (p < 0.01).

### 3.2 Biomechanical impact of trabecular-implant spatial relationship and optimal cephalocervical fixation positioning

**FIGURE 5:**
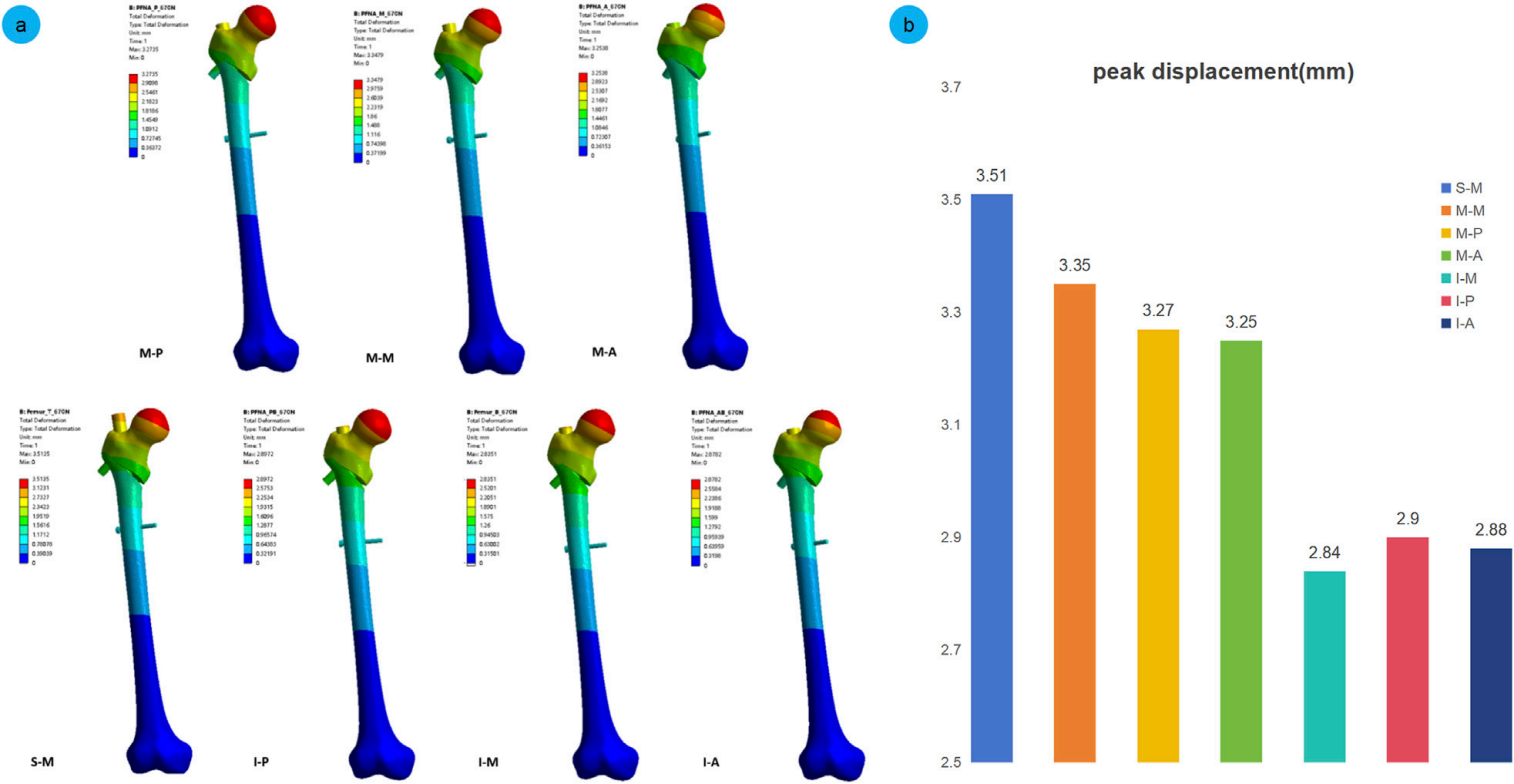
Femoral displacement distribution in finite element analysis **(a)** and comparison of peak displacement across groups **(b)**.

**FIGURE 6:**
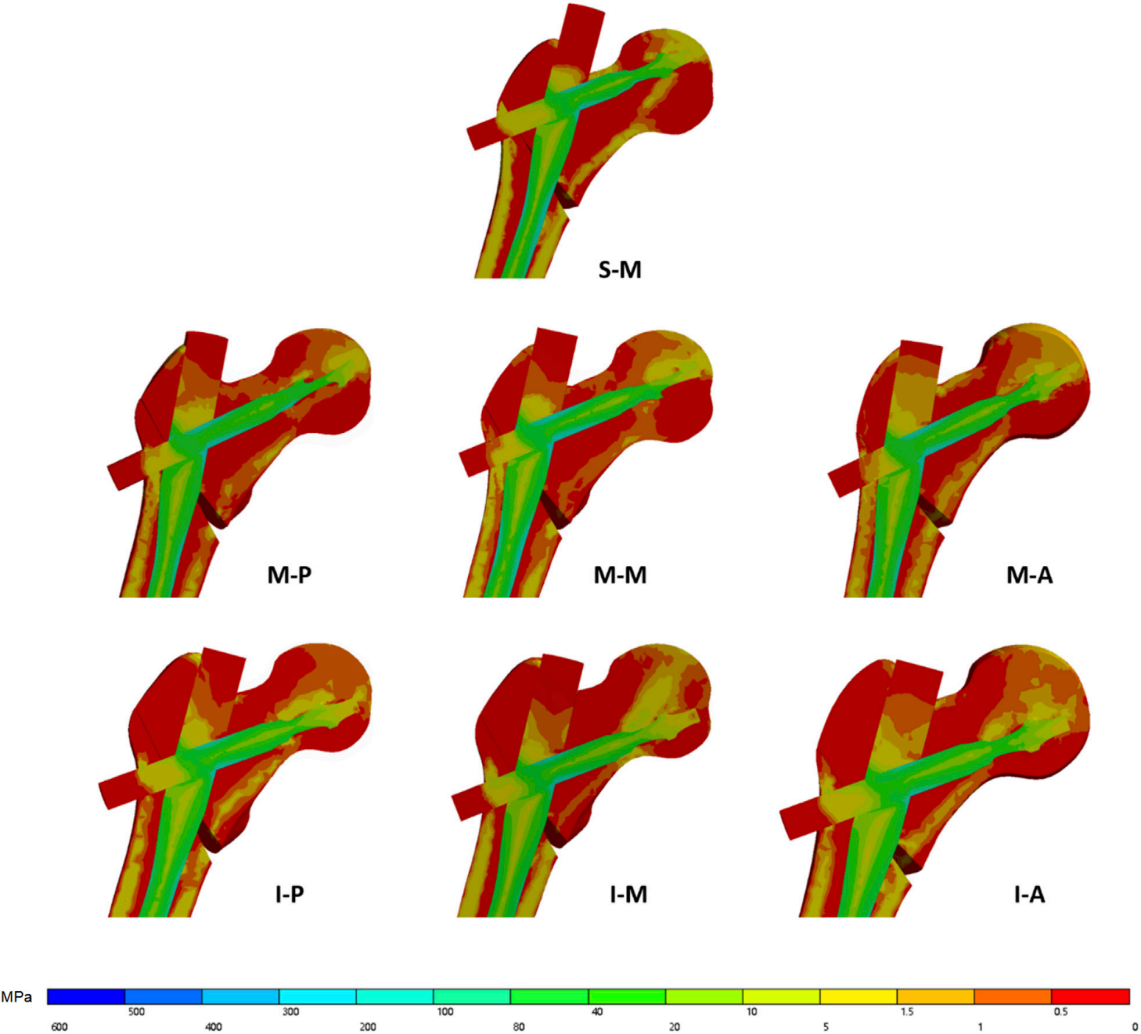
Finite element analysis of femoral coronal plane stress distribution.

The maximum displacement across all models consistently occurred at the load application site on the femoral head apex. In the coronal plane, inferior configurations (I-M, I-A, I-P) demonstrated significantly higher displacement values compared to middle and superior groups, with the inferior-medial (I-M) configuration exhibiting the smallest peak displacement—a reduction of 17.5% compared to superior-medial configurations (Figure 5). Additionally, stress distribution analysis revealed that the inferior positioning minimized stress concentration at the helical blade tip-cancellous bone interface. Instead, stress was predominantly distributed at the compressive trabecular bone-helical blade interface (Figure 6). These biomechanical observations align with our clinical retrospective analysis, confirming the critical role of the spatial relationship between compressive trabecular bone and the helical blade in enhancing fixation stability.

## 4 Discussion

This study assessed the influence of the spatial relationship between compressive trabecular bone and cephalocervical implants on immediate postoperative stability in intertrochanteric fractures. The findings indicated that an increased trabecular-implant lever arm constitutes an independent risk factor for femoral head varus and helical blade displacement. Positioning the helical blade within Zone C of the compressive trabecular architecture was correlated with reduced femoral head varus and implant displacement. Biomechanical analysis further demonstrated that implantation in the mid-inferior region of the femoral head (corresponding to Zone C) resulted in the least femoral head varus and blade displacement, with minimal stress concentration at the blade tip-cancellous bone interface. These results are consistent with the clinical retrospective analysis, underscoring the biomechanical importance of optimizing the compressive trabecular-implant spatial relationship.

Intertrochanteric fractures are predominantly addressed through early surgical intervention (Fischer et al., 2021). Even among patients with comorbid conditions such as renal insufficiency, diabetes, and ischemic heart disease—conditions that significantly affect in-hospital mortality—early hip surgery has been shown to positively influence survival rates (Rozenfeld et al., 2021). Nevertheless, postoperative implant failure frequently necessitates revision surgery, which results in increased overall costs, extended hospital and intensive care unit stays, greater transfusion requirements, higher infection rates, and increased mortality within the first year (Özdemir et al., 2021). Literature indicates that cephalocervical implant cutout, a significant mode of fixation failure, occurs in 5.8% of cases (Özdemir et al., 2021), with risk factors including bone quality, fracture type, reduction accuracy, and implant selection and positioning. Therefore, the optimal intraoperative placement of cephalocervical implants is essential. While conventional parameters like the tip-apex distance (TAD) and femoral head zoning are widely adopted in clinical practice (Haidukewych, 2010), their validity remains controversial. For instance, studies (Li et al., 2016; Subasi et al., 2022) employing computational modeling have proposed that TAD values are significantly influenced by femoral head size and may exhibit threshold limitations. The observation that inferior implant placement reduces cutout rates (De Bruijn et al., 2012) further challenges TAD’s reliability, this led to the proposal of CalTAD (Kuzyk et al., 2012). Our clinical retrospective analysis revealed that traditional parameters [TAD, CalTAD, Parker ratio (AP)], and the novel ABA correlate with femoral head collapse and implant displacement but do not independently predict these outcomes. Emerging debates highlight that isolated radiographic metrics may inadequately guide implant positioning, necessitating exploration of anatomical and biomechanical determinants. Based on anatomical and biomechanical principles, we propose that an increased trabecular-implant lever arm independently elevates risks of femoral head varus and blade migration. Conversely, positioning the implant within Zone C of the compressive trabecular architecture significantly reduces these complications, potentially serving as an intraoperative guide to optimize biomechanical stability.

In studies of cancellous bone distribution, researchers (Uemura et al., 2018) quantified regional cancellous bone density based on proximal femoral anatomical characteristics, though direct evidence linking heterogeneous cancellous distribution to stability outcomes remains lacking. Compressive trabecular bone is universally recognized as the primary load-bearing structure in the proximal femur. A biomechanical study by researchers (Liang et al., 2018) hypothesized that variations in the trabecular-implant spatial relationship theoretically alter load transfer patterns and stress distribution, yet conclusive evidence remains absent. Building upon these foundational studies, we propose the following hypothesis: The spatial relationship between compressive trabecular bone and cephalocervical implants critically modulates the postoperative biomechanical environment in intertrochanteric fracture fixation.

**FIGURE 7:**
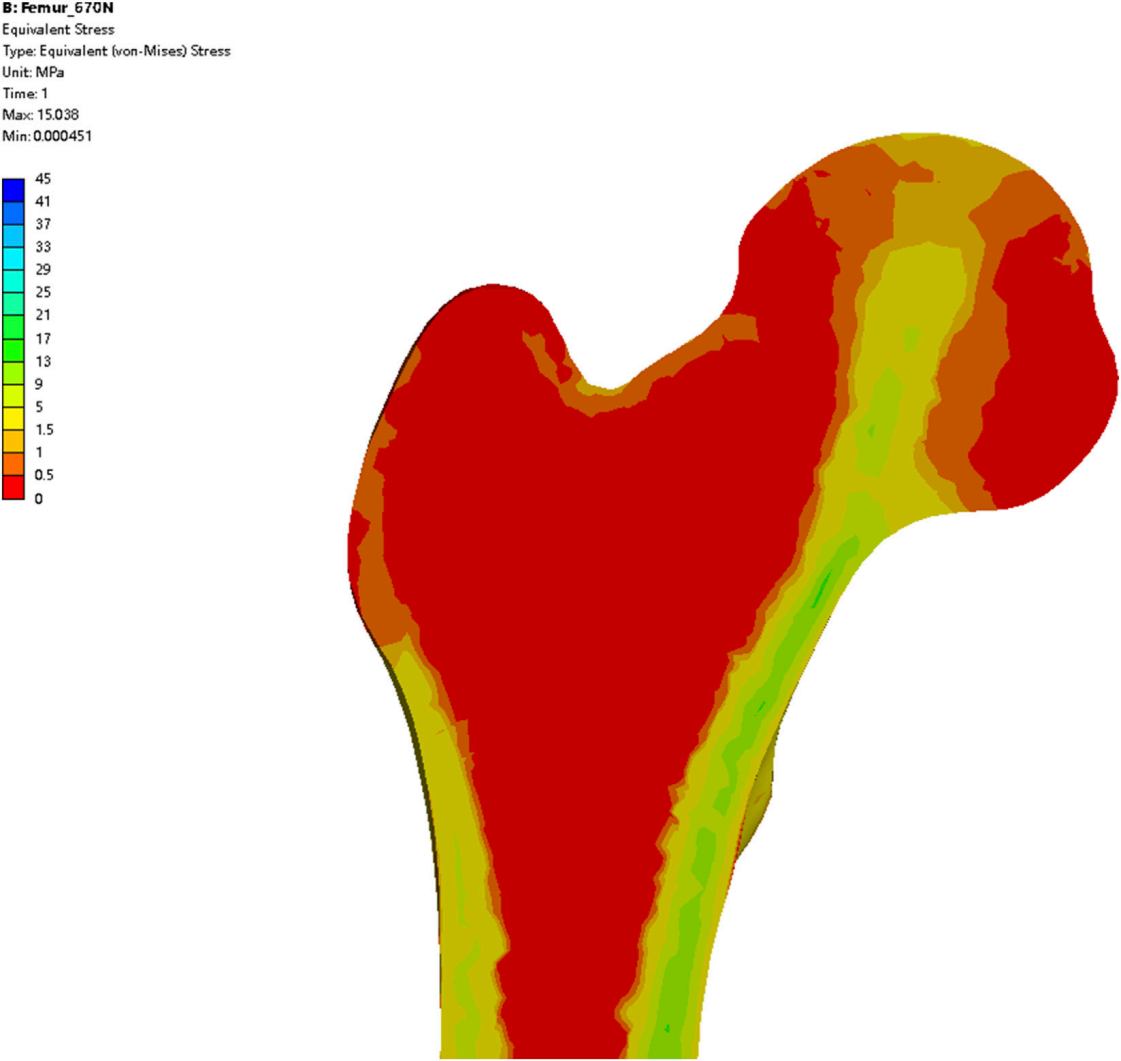
Von Mises stress distribution under physiological loading (670 N), demonstrating load transfer patterns through the compressive trabecular system.

Conventional tip-apex distance (TAD) criteria advocate positioning the cephalocervical implant centrally within the femoral head (mid-central zone), while calcar-referenced TAD (CalTAD) further emphasizes inferior-central placement. Existing femoral head zoning systems exhibit conflicting recommendations between mid-central and mid-inferior zones. In our study, Zone B and Zone C of the compressive trabecular architecture correspond to the mid-central and mid-inferior groups in traditional zoning systems. Final results demonstrated that Zone C achieved minimal peak displacement and reduced stress concentration at the implant tip, consistent with findings by Lee and Yang (Lee et al., 2018; Yang A. L. et al., 2023), though conflicting studies (De Bruijn et al., 2012) still favor mid-central positioning. Critical analysis reveals that earlier finite element studies predominantly employed a cortical-cancellous dichotomy for material property assignment—assigning uniform elastic moduli and Poisson’s ratios to cortical and cancellous bone separately. This approach fails to account for the heterogeneous density distribution of cancellous bone, particularly the biomechanical role of compressive trabeculae, potentially contributing to result discrepancies. In contrast, CT grayscale-based material mapping reconstructs the intrinsic heterogeneity of cancellous bone density. Validated in study (Zhang et al., 2013), this method has become standard in contemporary proximal femoral finite element analys (Yang Y. F. et al., 2023). Prior research (Zhang et al., 2023) utilized finite element analysis to map proximal femoral stress distribution under varying loads. Through principal stress visualization, these studies established a direct correlation between external mechanical stimuli and trabecular architecture: principal stress trajectories align with trabecular orientation, while trabecular density inversely correlates with absolute stress magnitudes. In our study, CT grayscale-based material assignment enabled physiological simulation of load transfer, with stress concentrated along compressive trabecular pathways (Figure 7), confirming the model’s fidelity in replicating *in vivo* biomechanical behavior.

Beyond the debate over mid-medial versus inferior-medial cephalocervical implant positioning, clinically unavoidable variations include superior-medial (S-M), mid-posterior (M-P), mid-anterior (M-A), inferior-posterior (I-P), and inferior-anterior (I-A) placements, all of which were simulated in our finite element analysis. Under transverse plane medial positioning, coronal plane superior configurations exhibited significantly greater femoral peak displacement and stress concentration at the implant tip, consistent with findings by Goffin (Goffin et al., 2013). Regarding sagittal plane positioning (comparisons among M-P, M-M, and M-A groups; and I-P, I-M, and I-A groups), current consensus (Goffin et al., 2013; Kane et al., 2014) suggests optimal placement in the mid-medial or inferior-medial zones. However, some studies (Caruso et al., 2022)propose that inferior-posterior or mid-posterior positioning may enhance cutout resistance. Our results showed no significant differences in maximum displacement across anterior, middle, and posterior zones. However, stress distribution analysis revealed more concentrated stress at the implant tip in anterior configurations compared to posterior placements, with minimal stress concentration observed in medial configurations.

Our study investigates cephalocervical implant positioning through the biomechanical lens of compressive trabecular bone anatomy, proposing that the trabecular-implant lever arm and zonal classification provide intraoperative guidance for optimal placement. The spatial relationship between compressive trabecular bone and implants further explains the rationale behind existing stability assessment methods. For instance, the calcar-referenced TAD (CalTAD) (Kuzyk et al., 2012), an improvement over conventional TAD, utilizes an inferior calcar reference line to better predict cutout risks, while femoral head zoning systems emphasize inferior positioning despite lacking anatomical justification. By integrating compressive trabecular anatomy—where trabeculae originate from the lesser trochanter and calcar to intersect tensile trabeculae in the femoral head—we demonstrate that inferior positioning at equivalent insertion depths reduces the trabecular-defined lever arm and positions the implant tip within Zone C. The recently proposed axis-blade angle (ABA) (Yang A. L. et al., 2023) identifies −10° as a critical threshold, with values below this sharply increasing complication risks. When ABA ≥ -10°, superior or anterior angulation relative to the femoral neck axis should be avoided—a principle corroborated by our data showing enhanced stability in intertrochanteric fractures with ABA ≥ -10° (P < 0.05). Anatomically, superior or upward-angled implants predispose placement in Zones A/B defined by compressive trabeculae, increasing the trabecular-defined lever arm and thereby compromising stability.

Based on these findings, we conclude that optimal initial fixation stability is achieved by positioning the cephalocervical implant within Zone C (corresponding to the inferior position in the coronal plane), followed by Zone B (mid-coronal position), while placement in Zone A (superior coronal position) should be avoided. In the sagittal plane, mid-positioning provides the greatest stability, followed by posterior placement, with anterior positioning demonstrating the poorest biomechanical performance. This approach may optimize the biomechanical environment for proximal femoral fixation, reconcile controversies in existing implant evaluation methods, and potentially enhance clinical outcomes.

However, our research also has limitations: 1. Measurement Variability: Despite standardized radiographic positioning, potential measurement inaccuracies in hip X-ray analyses cannot be entirely excluded. 2. Clinical cohort profile: This single-center retrospective cohort study included 62 cases with limited follow-up duration. We recommend future prospective multicenter studies featuring extended follow-up periods to further investigate potential nonlinear associations. 3. Model Demographics: The finite element model utilized CT data from a 20-year-old male, whereas intertrochanteric fractures predominantly occur in osteoporotic populations.Although the current model did not explicitly incorporate osteoporotic parameters, the compressive trabeculae, constituting the core structural element of the key load-transfer pathway in the proximal femur, remain present and functionally relevant in the elderly population (Uemura et al., 2018). Consequently,the stability advantage associated with Zone C implantation observed in this study may exhibit generalizability. Future studies incorporating osteoporotic specimens for FE analysis or biomechanical testing are warranted to further validate this inference. Furthermore, exclusion of musculature modeling and dynamic gait cycles - a biomechanically essential element - constitutes an experimental limitation potentially affecting outcome validity; consequently, dedicated verification through muscle-integrated simulations is mandated in subsequent research. 4. Model Fidelity: Although CT grayscale-based modeling reduces discrepancies between simulated and actual bone properties, inherent differences between computational models and *in vivo* biomechanics persist.

## 5 Conclusion

The spatial relationship between compressive trabecular bone and cephalocervical implants provides intraoperative positional guidance and provides anatomical elucidation of prior implant stability assessment methodologies. During helical blade insertion, surgeons may need to reference compressive trabecular anatomy to shorten the trabecular-blade lever arm length and optimally position the blade within Zone C of the trabecular architecture to achieve enhanced early stability.

## Data Availability

The original contributions presented in the study are included in the article/supplementary material, further inquiries can be directed to the corresponding authors.

## References

[B1] Adeyemi A. Delhougne G. (2019). Incidence and economic burden of intertrochanteric fracture: a medicare claims database analysis. JB and JS open access 4 (1), e0045. 10.2106/JBJS.OA.18.00045 31161153 PMC6510469

[B2] Baumgaertner M. R. Curtin S. L. Lindskog D. M. Keggi J. M. (1995). The value of the tip-apex distance in predicting failure of fixation of peritrochanteric fractures of the hip. J. bone Jt. Surg. Am. volume 77 (7), 1058–1064. 10.2106/00004623-199507000-00012 7608228

[B3] Caruso G. Corradi N. Caldaria A. Bottin D. Lo Re D. Lorusso V. (2022). New tip-apex distance and calcar-referenced tip-apex distance cut-offs May be the best predictors for cut-out risk after intramedullary fixation of proximal femur fractures. Sci. Rep. 12 (1), 357. 10.1038/s41598-021-04252-1 35013492 PMC8748913

[B4] Çepni Ş. Subaşı İ. Ö. Şahin A. Bozkurt İ. Fırat A. Kılıçarslan K. (2022). Tip-neck distance ratio as a novel predictor for failure in cephalomedullary nailing of unstable trochanteric fractures (UTF). Archives Orthop. trauma Surg. 142 (10), 2619–2626. 10.1007/s00402-021-03999-6 34146115

[B5] Cheung C. L. Ang S. B. Chadha M. Chow E. S. Chung Y. S. Hew F. L. (2018). An updated hip fracture projection in Asia: the Asian Federation of osteoporosis societies study. Osteoporos. sarcopenia 4 (1), 16–21. 10.1016/j.afos.2018.03.003 30775536 PMC6362950

[B6] Cleveland M. Bosworth D. M. Thompson F. R. Wilson H. J. Jr Ishizuka T. (1959). A ten-year analysis of intertrochanteric fractures of the femur. J. bone Jt. Surg. Am. 41-A, 1399–1408. 10.2106/00004623-195941080-00003 13849408

[B7] De Bruijn K. den Hartog D. Tuinebreijer W. Roukema G. (2012). Reliability of predictors for screw cutout in intertrochanteric hip fractures. J. bone Jt. Surg. Am. volume 94 (14), 1266–1272. 10.2106/JBJS.K.00357 22810396

[B8] Eberle S. Gerber C. von Oldenburg G. Högel F. Augat P. (2010). A biomechanical evaluation of orthopaedic implants for hip fractures by finite element analysis and *in-vitro* tests. Proc. Institution Mech. Eng. Part H, J. Eng. Med. 224 (10), 1141–1152. 10.1243/09544119JEIM799 21138232

[B9] Fischer H. Maleitzke T. Eder C. Ahmad S. Stöckle U. Braun K. F. (2021). Management of proximal femur fractures in the elderly: current concepts and treatment options. Eur. J. Med. Res. 26 (1), 86. 10.1186/s40001-021-00556-0 34348796 PMC8335457

[B10] Garabano G. Juri A. Perez Alamino L. Rodriguez J. A. Pesciallo C. A. (2024). Predicting cut-out in intertrochanteric fractures fixed with cephalomedullary nails: the role of tip-to-apex distance referenced to calcar (calTAD)--A retrospective analysis of 158 cases. Eur. J. Orthop. Surg. Tr. 35 (1), 24. 10.1007/s00590-024-04130-2 39585420

[B11] Goffin J. M. Pankaj P. Simpson A. H. (2013). The importance of lag screw position for the stabilization of trochanteric fractures with a sliding hip screw: a subject-specific finite element study. J. Orthop. Res. official Publ. Orthop. Res. Soc. 31 (4), 596–600. 10.1002/jor.22266 23138576

[B12] Haidukewych G. J. (2010). Intertrochanteric fractures: ten tips to improve results. Instr. course Lect. 59, 503–509. 20415401

[B13] Kane P. Vopat B. Heard W. Thakur N. Paller D. Koruprolu S. (2014). Is tip apex distance as important as we think? A biomechanical study examining optimal lag screw placement. Clin. Orthop. Relat. Res. 472 (8), 2492–2498. 10.1007/s11999-014-3594-x 24760583 PMC4079854

[B14] Kuzyk P. R. Zdero R. Shah S. Olsen M. Waddell J. P. Schemitsch E. H. (2012). Femoral head lag screw position for cephalomedullary nails: a biomechanical analysis. J. Orthop. trauma 26 (7), 414–421. 10.1097/BOT.0b013e318229acca 22337483

[B15] Lee C. H. Su K. C. Chen K. H. Pan C. C. Wu Y. C. (2018). Impact of tip-apex distance and femoral head lag screw position on treatment outcomes of unstable intertrochanteric fractures using cephalomedullary nails. J. Int. Med. Res. 46 (6), 2128–2140. 10.1177/0300060518775835 29848122 PMC6023058

[B16] Li J. T. (2016). Finite element analysis of proximal femoral nail antirotation (PFNA) and medial support nail (MSN) in the treatment of intertrochanteric fractures. Chin. PLA Med. Coll.

[B17] Li S. Chang S. M. Jin Y. M. Zhang Y. Q. Niu W. X. Du S. C. (2016). A mathematical simulation of the tip-apex distance and the calcar-referenced tip-apex distance for intertrochanteric fractures reduced with lag screws. Injury 47 (6), 1302–1308. 10.1016/j.injury.2016.03.029 27087281

[B18] Liang C. Peng R. Jiang N. Xie G. Wang L. Yu B. (2018). Intertrochanteric fracture: association between the coronal position of the lag screw and stress distribution. Asian J. Surg. 41 (3), 241–249. 10.1016/j.asjsur.2017.02.003 28366494

[B20] Mao W. He Y. Q. Tang H. Chen X. J. Li L. L. Dong Y. H. (2019). A novel angle on helical blade placement in trochanteric fractures - the axis-blade angle. Injury 50 (7), 1333–1338. 10.1016/j.injury.2019.05.006 31130219

[B21] Nie S. Li J. Li M. Hao M. Wang K. Xiong Y. (2022). Finite-element analysis of a novel cephalomedullary nail for restricted sliding to reduce risk of implant failure in unstable intertrochanteric fractures. Orthop. Surg. 14 (11), 3009–3018. 10.1111/os.13497 36120825 PMC9627085

[B22] Özdemir E. Okkaoglu M. C. Evren A. T. Yaradilmis Y. U. Ates A. Altay M. (2021). The cost and consequences of failed osteosynthesis of intertrochanteric femur fractures: a matched cohort study. Indian J. Orthop. 55 (3), 629–635. 10.1007/s43465-020-00322-0 33995866 PMC8081792

[B23] Parker M. J. (1992). Cutting-out of the dynamic hip screw related to its position. J. bone Jt. Surg. Br. volume 74 (4), 625. 10.1302/0301-620X.74B4.1624529 1624529

[B24] Ren Y. Hu J. Lu B. Zhou W. Tan B. (2019). Prevalence and risk factors of hip fracture in a middle-aged and older Chinese population. Bone 122, 143–149. 10.1016/j.bone.2019.02.020 30797059

[B25] Rozenfeld M. Bodas M. Shani M. Radomislensky I. Murad H. Comaneshter D. (2021). National study: most elderly patients benefit from earlier hip fracture surgery despite co-morbidity. Injury 52 (4), 905–909. 10.1016/j.injury.2020.10.060 33082028

[B26] Subasi O. Aslan L. Demirhan M. Seyahi A. Lazoglu I. (2022). A novel lower bound for tip-apex distance. Eur. J. trauma Emerg. Surg. official Publ. Eur. Trauma Soc. 48 (3), 1787–1798. 10.1007/s00068-020-01514-x 33037920

[B27] Uemura K. Takao M. Otake Y. Hamada H. Sakai T. Sato Y. (2018). The distribution of bone mineral density in the femoral heads of unstable intertrochanteric fractures. J. Orthop. Surg. (Hong Kong) 26 (2), 2309499018778325. 10.1177/2309499018778325 29852815

[B28] Wu L. F. Zhang T. S. Li J. Huang H. Zhou C. H. Li X. (2024). Construction and validation of a nomogram prediction model for internal fixation failure after proximal femoral anti-rotation intramedullary nailing in the treatment of intertrochanteric fractures of the femur. Med. Baltim. 103 (47), e40575. 10.1097/MD.0000000000040575 39809193 PMC11596523

[B29] Yang Y. F. Huang J. W. Gao X. S. Xu Z. H. (2023a). Standardized tip-apex distance (STAD): a modified individualized measurement of cephalic fixator position based on its own femoral head diameter in geriatric intertrochanteric fractures with internal fixation. BMC Musculoskelet. Disord. 24 (1), 189. 10.1186/s12891-023-06286-0 36915071 PMC10009924

[B30] Yang A. L. Mao W. Chang S. M. He Y. Q. Li L. L. Long F. (2023b). Computational evaluation of the axis-blade angle for measurements of implant positions in trochanteric hip fractures: a finite element analysis. Comput. Biol. Med., 158, 106830. 10.1016/j.compbiomed.2023.106830 37011432

[B31] Zhang Y. Zhong W. Zhu H. Chen Y. Xu L. Zhu J. (2013). Establishing the 3-D finite element solid model of femurs in partial by volume rendering. Int. J. Surg. Lond. Engl. 11 (9), 930–934. 10.1016/j.ijsu.2013.06.843 23832095

[B32] Zhang J. Li H. Zhou Y. Chen S. Rong Q. (2023). An analysis of trabecular bone structure based on principal stress trajectory. Bioeng. Basel, Switz. 10 (10), 1224. 10.3390/bioengineering10101224 37892954 PMC10604682

